# Neuroprotective Effect of Benzyl Ferulate on Ischemia/Reperfusion Injury via Regulating NOX2 and NOX4 in Rats: A Potential Antioxidant for CI/R Injury

**DOI:** 10.1155/2024/5534135

**Published:** 2024-11-02

**Authors:** Yu Xiang, Li Mao, Zhao-Hui Dai, Xiao-Hua Liu, Zhong-Bao Yang

**Affiliations:** ^1^Chest Pain Center of Changsha, The Affiliated Changsha Hospital of Hunan Normal University, Changsha 410006, Hunan, China; ^2^Department of Basic Medicine, Changsha Health Vocational College, Changsha 410600, Hunan, China

**Keywords:** benzyl ferulate, cerebral ischemia/reperfusion injury, NOX2, NOX4, oxidative stress

## Abstract

Oxidative stress is a primary contributor to cerebral ischemia/reperfusion (CI/R) injury, and the use of antioxidants represents a crucial therapeutic strategy for managing CI/R injury. This study aims to explore the antioxidant effects of benzyl ferulate on CI/R injury and elucidate its underlying mechanisms. In vivo models of CI/R injury and hypoxia/reoxygenation (H/R) injury in SH-SY5Y cells were established, followed by treatment with benzyl ferulate. The extent of oxidative stress was assessed through evaluations of neurobiological function, cerebral infarct volume, reactive oxygen species (ROS), apoptosis levels, etc. Results indicated that benzyl ferulate significantly downregulated the expression of NADPH oxidase 2 (NOX) 2/NOX4 while upregulating miRNAs (652/532/92b) in CI/R rats or SH-SY5Y cells. It also reduced total NOX enzyme activity, enhanced superoxide dismutase (SOD) activity, decreased ROS and malondialdehyde (MDA) production, and inhibited cleaved caspase-3 and Bax expression—ultimately leading to reduced cell apoptosis. Benzyl ferulate effectively mitigates oxidative stress injuries of middle cerebral artery occlusion (MCAO) rats or SH-SY5Y cells subjected to H/R, and its mechanism appears to involve modulation of the miRNAs (652/532/92b)/NOX2/4 axis. This study first proved that benzyl ferulate is a promising antioxidant candidate for treating CI/R injury.

## 1. Introduction

Oxidative stress is one of the leading causes that lead to cerebral ischemia/reperfusion (CI/R) injury [1]. Recently, restoring blood circulation by surgery or thrombolysis using drugs (rt-PA) within the therapeutic window of ischemic stroke is the effective intervention for ischemic stroke [2]. However, timely blood recovery shortly will cause damage to the neurons, which is CI/R injury [3]. Numerous studies have confirmed that the imbalance of oxygen production and scavenging of the body or cells would lead to reactive oxygen species (ROS) overproduction, which results in cell injury by damaging the function of macromolecules, such as nucleic acids, proteins, and enzymes [1]. The reported studies have demonstrated that high-expressed enzymes that are responsible for ROS production, such as NADPH oxidase (NOX) and xanthine oxidase, which associated with oxidative stress during the process of CI/R injury [1]. NOX was widely accepted as a contributor to accelerating tissue attacks, which were extensively studied for CI/R injury [4]. Therefore, antioxidant that targeting NOX would be effective for CI/R injury. It was reported that NOX2 inhibitor exerts neuroprotection in experimental studies, such as apocynin, which can decrease the brain infarct size of middle cerebral artery occlusion (MCAO) rats and improve its neurological function [5]. Until now, to our knowledge, no such available inhibitors were used clinically nowadays, which suggested that more works need to be done to elucidate the regulating mechanism of NOX2 in CI/R injury or finding its novel inhibitor.

miRNAs are a class of noncoding short RNAs with a length of 22 nt and play important roles in gene expression by binding to the 3′UTR of mRNA in a complementary or incomplete complementary manner [6]. Our and other previous studies have found that miR-652, miR-532, and miR-92b play pivotal roles in CI/R injury by directly targeting NOX2 or NOX4 [7, 8]. Considering that the abnormal expression of miR-652, miR-532, and miR-92b was closely associated with CI/R injury, we speculate that these miRNAs may be potential targets for screening drugs with antioxidation effect.

Ferulic acid is an active compound that extracted from *Ligusticum chuanxion*g. Large numbers of studies have demonstrated that ferulic acid possessed potent antioxidant capacities [9]. Ferulic acid could scavenge oxygen radical and protect rats from CI/R injury by decreasing brain infarct size and improving neurological function [9, 10]. However, due to its low lipid solubility and cannot easily penetrate the blood–brain barrier [11], the effect of ferulic acid in clinic is poor. Studies have demonstrated that ferulic acid undergoes xenobiotic metabolism in the human body and forms derivatives through sulfonation or conjugation with glucuronic acid, and the majority of them are excreted in the urine [12]. Hence, an extremely small amount of ferulic acid can enter into the bloodstream and fewer can cross blood–brain barrier into brain tissue. Due to this, the bioavailability of ferulic acid is low. Thus, increasing the lipid solubility of ferulic acid may improve its neuroprotective role. Benzyl ferulate is the esters of ferulic acid, with higher lipid solubility than ferulic acid theoretically. Therefore, we speculate that benzyl ferulate is a potential antioxidant for CI/R injury.

This study was intended to investigate the neuroprotection of benzyl ferulate and its underlying mechanism.

## 2. Materials and Methods

### 2.1. Animal Experiment

Male SD rats (250–290 g of weight, 8 weeks old) were used for experiments. All the animals were provided by a commercial company (Hunan SJA Laboratory Animal Co., Ltd.). Before or during the process of experiments, the animals were housed in conditions as follows: Environmental temperature and humidity were 25°C and 65%, respectively, and every 12-h light followed by a 12-h dark. The experiment was approved by the Ethics Committee of the Affiliated Changsha Hospital of Hunan Normal University.

To mimic ischemic stroke, a MCAO model was established as previously reported by Mao et al. [7]. The rats were lightly anesthetized by intraperitoneally injected with pentobarbital sodium (3%) with the dosage of 40 mg/kg body weight. Followed that, the hairs on the lower jaw of rats were removed and the skin of the neck was exposed and disinfected using iodine tincture. Then, the skin of the neck was incised using scissors and isolated the rat's left internal carotid artery. Then, the artery was occluded by inserting a rounded tip surgical nylon monofilament (4/0) and the blood flow to the brains was terminated. After 2 h of ischemia, the filament was removed and followed by a 24-h reperfusion of blood. During the whole surgical course, the vital signs of animals were detected every 30 min to make sure its body temperature and respiratory rate are normal; for example, body temperature maintains at 37°C. The sham group only incised the skin but not insert the occluding filament.

Animals were randomly assigned into six groups (*n* = 6 per group): sham group (without ischemic insult), I/R group (the rats subjected to a 2-h ischemia followed by a 24-h reperfusion), I/R + BF (L) group (low-dose benzyl ferulate expose group: The I/R rats were administrated with 5 mg/kg body weight benzyl ferulate), I/R + BF (M) group (mid-dose benzyl ferulate expose group: The I/R rats were administrated with 10 mg/kg body weight benzyl ferulate), I/R + BF (H) group (high-dose benzyl ferulate expose group: The I/R rats were administrated with 20 mg/kg body weight benzyl ferulate), and the vehicle group (the I/R rats were administrated with saline). The rats were administrated with benzyl ferulate by gavage at the initial of surgery. Followed the end of 24 h of reperfusion, the neurobiological function was evaluated following the methods by Mao et al. [7]. At the end, the rats were killed by behead and the brain tissues were collected.

### 2.2. Cell Culture

The SH-SY5Y cell line was provided by Central South University and authenticated by STR profiling. Cells hold in standard conditions as follows: at 37°C, 95% air and 5% CO2. DMEM medium that supplements with 10% FBS and penicillin/streptomycin was used for cell standard culture. For drug intervention, SH-SY5Y cells were seeded into 12-well plates. At the end, cells were collected for further analysis.

### 2.3. Cell Hypoxia/Reoxygenation (H/R) Model

To simulate ischemia stroke in vitro, the SH-SY5Y cells were subjected to a 4 h of hypoxia followed by a 20-h reoxygenation. During the hypoxia process, the cells were incubated with Dulbecco's phosphate-buffered saline (DPBS) and the condition of cell incubator was changed to 95% N2, 5% CO_2_. During the reoxygenation, the cells were incubated with standard compound cell culture medium and the condition of cell incubator was adjusted to 95% air and 5% CO2.

The cells were grouped as follows: the control group, the H/R group (cells insulted with a 4-h hypoxia followed by a 20-h reoxygenation), H/R + BF group (cells subjected to H/R were treated with different dosage of BF: 10^−8^ M, 10^−7^ M, and 10^−6^ M), and the vehicle group (cells subjected to H/R were treated with DMSO).

### 2.4. Assessment of Neurological Deficit Score

The rat neurological function was assayed using a five-point rating scale. According to the scale, the value 0 means no deficit, the value 1 means failure to extend the left forepaw, the value 2 means decreased grip strength of left forepaw, the value 3 means circling to the left on pulling the tail, and the value 4 means spontaneous circling.

### 2.5. Measurement of Infarct Volume

The brain infarct was observed following the methods by Mao et al. [7]. Briefly, brains were frozen for 20 min in a −20°C refrigerator and then sectioned into 4 coronal sections, each with a thickness of 0.2–0.3 cm. After that, the brain sections were incubated with 2% 2,3,5-tripenyltetrazolium chloride (TTC) for 30 min in dark at 37°C. Followed that, the brain sections were imaged for infarct volume analysis. The infarct volume (cm^3^) was calculated as follows:

(1) $$\begin{array}{c} \text{total infarct volume}=\text{the sum of infarct volumes of all sections }\left(\text{infarct volume of each section equals infarct area }\left({\text{cm}}^{2}\right)\text{ timed by its thickness}\right). \end{array}$$

### 2.6. NOX and Superoxide Dismutase (SOD) Enzymatic Activity Determination

The NOX enzyme activity was measured using a commercial kit (Genmed Pharmaceutical Technology Co., Ltd., Shanghai, China). Briefly, the cells or tissue lysates were centrifuged (4000r/ min, 10 min) and the supernatant was collected for enzyme reaction analysis. A reaction solution (made up of cell lysates, oxidized cytochrome C, and NADPH) was established and held it into a quartz cuvette for 15 min. Spectrophotometry was used for the absorbance of the reaction solution at 550 nm. A unit of NOX enzyme activity was defined as the reduction of cytochrome C per min.

The total SOD activities were detected using a commercial kit (Beyotime, China, S0101). Briefly, the cells were collected and fully lysed using the RIPA buffer first. Then, the lysate was centrifuged at 1000g at 4°C for 10 min and the supernatant was collected for total SOD activity measurements. OD450 was read with microplate reader (BioTek ELx800, USA). The SOD activity was expressed as enzyme unit/mg.

### 2.7. Measurement of Malondialdehyde (MDA) Contents

Thiobarbituric acid (TBA) method was used for MDA content determination. Briefly, cells or tissues were fully lysed and its supernatant was collected for the establishment of reaction mixture (made up of cell lysates, phosphoric acid, and 0.67% TBA). For fully reaction, the mixture was heated at 95°C for 60 min. Then, 375 mL of *N*-butanol was added into the mixture and mixed up fully. The upper *N*-butanol layer was transferred to a glass tube. The OD532 was read, and the MDA content was calculated by mmol/mg protein.

### 2.8. Cell Apoptosis Analysis

To assess the cell apoptosis, TUNEL staining was conducted with a commercial TUNEL assay kit (Beyotime, Shanghai, China). Briefly, tissues were fixed using 4% w/v paraformaldehyde at 25°C and then rinsed twice using PBS. Followed that, the tissues were postfixed using paraformaldehyde plus acetic acid at 4°C and then rinsed twice with PBS. After that, the tissues were treated with equilibration buffer and working strength deoxynucleotide transferase (TdT) successively. For fully reaction, the tissues were maintained at 37°C for 1 h. The apoptosis was observed by fluorescence microscope at ×200 magnification (Nikon Eclipse 80i). The cell apoptosis was expressed as TUNEL-positive cell percentage.

### 2.9. Flow Cytometry

Annexin V-FITC Apoptosis Detection Kit (Beyotime, China) was used for flow cytometry cell apoptosis analysis. First, the SH-SY5Y cells were suspended using PBS to make a cell suspension. Followed that, Annexin V-FITC and PI (propidium iodide) were added into the prepared cell suspension successively and mixed up. Then, the suspension was incubated at 25°C for 15 min in the dark. At the end, the cell apoptosis was assayed by flow cytometry (BD FACSCalibur, USA) and CellQuest Pro software.

### 2.10. Measurement of Cell Viability

The Cell Counting Kit-8 (CCK-8) was used for cell viability measurement. The OD490 was read using a microplate reader (BioTek ELx800, USA). According to the manufacturer's instruction, the OD values are proportional to the number of cells.

### 2.11. Determination of ROS Levels

A ROS assay kit (DCFH-DA, Beyotime, Shanghai, China) was used for total ROS level determination. Briefly, the SH-SY5Y cells were immersed with 10 mM DCFH-DA and put it at 37°C for 20 min. After that, the fluorescence intensity was detected using a fluorescent microscope (Leika). Arbitrary units are used for fluorescence intensity.

### 2.12. Determination of Ferulic Acid and Benzyl Ferulate Concentrations in Plasma and Brain Tissue by HLPC

SD rats were administered ferulic acid or benzyl ferulate (20 mg/kg body weight) via gavage. One hour after the administration, SD rats were anesthetized by injection of sodium pentobarbital and sacrificed, followed by the collection of plasma and brain tissue. The concentrations of ferulic acid and benzyl ferulate in plasma and brain tissue homogenates were determined by HLPC.

### 2.13. Real-Time PCR

Total RNA extraction was performed using the Trizol reagent kit (TakaRa Biotechnology Co., Ltd., Dalian, China). Briefly, cells or tissues were collected and mixed with Trizol to make a reaction mixture and placed it on ice for 25 min. Followed that, the mixture was centrifuged at 12000r/ min for 10 min and the RNA precipitate was collected and dissolved in sterile water. RNA purity and concentration of the extracted RNA solution were determined spectrophotometrically. Followed that, a 10-*μ*L reverse transcription reaction system and a 25-*μ*L real-time PCR system were used for cDNA production and RNA quantification, respectively. The ABI 7300 plus Real-Time PCR System was used for amplification reaction. The 2^−ΔΔCq^ method was used for data analysis, and results were expressed as the ratio of miRNA to U6 [13].

### 2.14. Western Blot

Total protein was extracted using a commercial kit (cell lysis buffer for Western and IP, Beyotime, Shanghai, China). Briefly, cells or tissues were collected and mixed with the cell lysis buffer to make a reaction mixture and placed it on ice for 20 min. After that, the cell lysate was centrifuged at 4000r/ min for 10 min and the total protein was obtained. Then, the protein was denaturized at 99°C. Western blot analysis was performed following the methods by Mao et al. [7]. SDS–PAGE was used for protein isolation. Briefly, preparation of a 10% gel and each lane of the gel was loaded 40 *μ*g of protein from different samples. Then, the target protein was isolated by electrophoresis and transferred to polyvinylidene fluoride membrane. Before incubating with primary antibody of NOX2, NOX4, Bcl2, Bax, Caspase-3, or *β*-actin (Santa Cruz Biotechnology, Santa Cruz, CA) at 4°C overnight, the membrane was treated with 20% fat-free milk. Then, membranes were treated with horseradish peroxidase-conjugated secondary antibodies at 25°C for 1 h. At the end, the band signal intensity was analyzed and densitometric value was normalized to *β*-actin.

### 2.15. Statistical Analysis

The SPSS 19 was used for statistical analysis. Data were expressed as mean ± SD. One-way analysis of variance was used to compare the data distribution between groups. Tukey's test was used for post hoc comparisons of specific groups. Differences were considered significant when *p* < 0.05.

## 3. Results

### 3.1. Benzyl Ferulate Attenuate CI/R Injury in Rat

**Figure 1:**
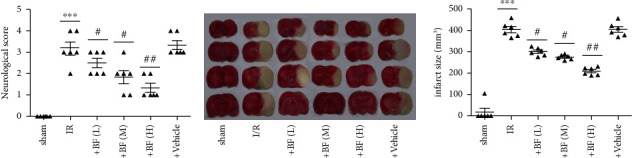
Benzyl ferulate attenuate CI/R injury in rat. (a) Neurological score; (b) TTC staining; (c) infarction volume differences of the values between groups were analyzed by one-way ANOVA. All experiments were repeated 3 times, and all data are presented as the mean ± standard deviation (*n* = 6), sham: rats just subjected to sham; I/R: rats subjected to 2-h ischemia followed by 24-h reperfusion; +BF (L): rats subjected to I/R treated with benzyl ferulate (5 mg/kg); +BF (M): rats subjected to I/R treated with benzyl ferulate (10 mg/kg); +BF (H): rats subjected to I/R treated with benzyl ferulate (15 mg/kg). ⁣^∗∗∗^*p* < 0.001 vs sham; ^#^*p* < 0.05 vs I/R; ^##^*p* < 0.01 vs I/R.

As shown in Figure 1(a), compared to the sham, obvious neurological function damage occurred in MCAO rats, while benzyl ferulate significantly improved the neurological function dose-dependently. The TTC staining showed that obvious brain infarction occurred in MCAO rats and benzyl ferulate significantly reduced the infarction size dose-dependently (Figures 1(b) and 1(c)).

### 3.2. Effect of Benzyl Ferulate on Cellular Apoptosis in the Brain of CI/R Injury Rats

**Figure 2:**
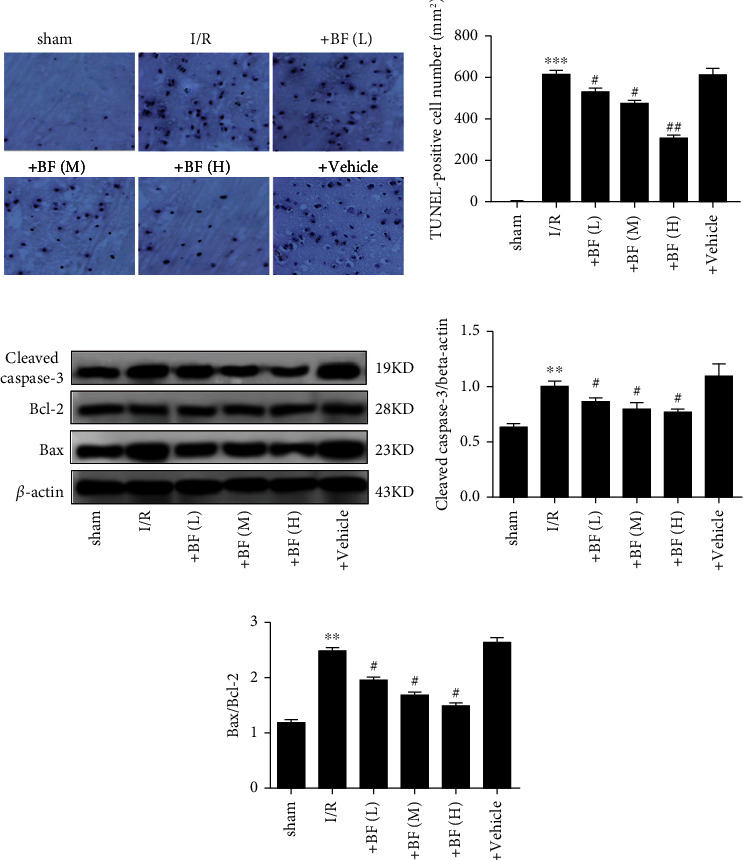
Effect of benzyl ferulate on cellular apoptosis in the brain of CI/R injury rats. (a) TUNEL staining; (b) TUNEL-positive cell number; (c) protein expression level; (d) the ratio of cleaved caspase-3 to beta-actin; (e) the ratio of Bax to Bcl-2. The differences of the values between groups were analyzed by one-way ANOVA. All experiments were repeated 3 times, and all data are presented as the mean ± standard deviation, sham: rats just subjected to sham; I/R: rats subjected to 2-h ischemia followed by 24-h reperfusion; +BF (L): rats subjected to I/R treated with benzyl ferulate (5 mg/kg); +BF (M): rats subjected to I/R treated with benzyl ferulate (10 mg/kg); +BF (H): rats subjected to I/R treated with benzyl ferulate (15 mg/kg). ⁣^∗∗^*p* < 0.01 vs sham; ⁣^∗∗∗^*p* < 0.001 vs sham; ^#^*p* < 0.05 vs I/R; ^##^*p* < 0.01 vs I/R.

TUNEL assay was performed to measure the apoptosis of neurons. Compared to the sham, the number of TUNEL-positive cells obviously increased in brains of MCAO rats, while administration of benzyl ferulate obviously reduced the number of TUNEL-positive cell dose-dependently (Figures 2(a) and 2(b)). The ratio of Bax/Bcl-2 and the expression of cleaved caspase-3 obviously increased in brain tissues of MCAO rats and benzyl ferulate significantly decreased the expression of these apoptotic proteins dose-dependently (Figures 2(c), 2(d), 2(e)).

### 3.3. Effect of Benzyl Ferulate on Oxidative Stress in the Brain of CI/R Injury Rats

**Figure 3:**
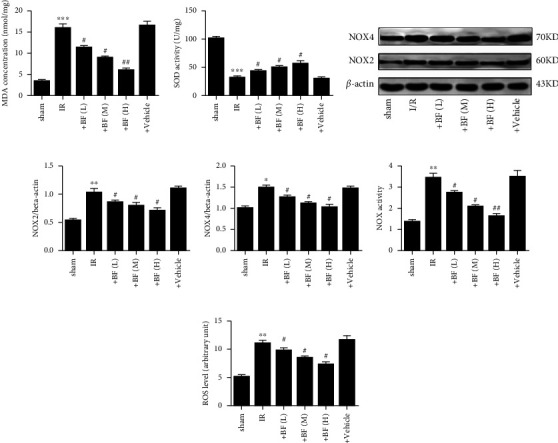
Effect of benzyl ferulate on oxidative stress in the brain of CI/R injury rats. (a) MDA content; (b) SOD activity; (c) protein expression level; (d) the ratio of NOX2 to beta-actin; (e) the ratio of NOX4 to beta-actin; (f) total NOX enzyme activity; (g) ROS level. The differences of the values between groups were analyzed by one-way ANOVA. All experiments were repeated 3 times, and all data are presented as the mean ± standard deviation. Sham: rats just subjected to sham; I/R: rats subjected to 2-h ischemia followed by 24-h reperfusion; +BF (L): rats subjected to I/R treated with benzyl ferulate (5 mg/kg); +BF (M): rats subjected to I/R treated with benzyl ferulate (10 mg/kg); +BF (H): rats subjected to I/R treated with benzyl ferulate (15 mg/kg). ⁣^∗^*p* < 0.05 vs sham; ⁣^∗∗^*p* < 0.01 vs sham; ⁣^∗∗∗^*p* < 0.001 vs sham; ^#^*p* < 0.05 vs I/R; ^##^*p* < 0.01 vs I/R.

Compared to the sham, the MDA content was obviously increased and the SOD activity obviously reduced in the brain of MCAO rats, while benzyl ferulate reduced the MDA content and increased the SOD activity in a dose-dependent manner (Figures 3(a) and 3(b)). The expression level of NOX2 and NOX4, the NOX activity, and the ROS level obviously increased in the brain of MCAO rats when compared with the sham, while benzyl ferulate dose-dependently reversed these changes by decreasing the expression of NOX2 and NOX4, the total NOX activity, and the ROS level (Figures 3(c), 3(d), 3(e), 3(f), 3(g)).

### 3.4. Effect of Benzyl Ferulate on Cellular Apoptosis of H/R-Treated SH-Y5Y Cells

**Figure 4:**
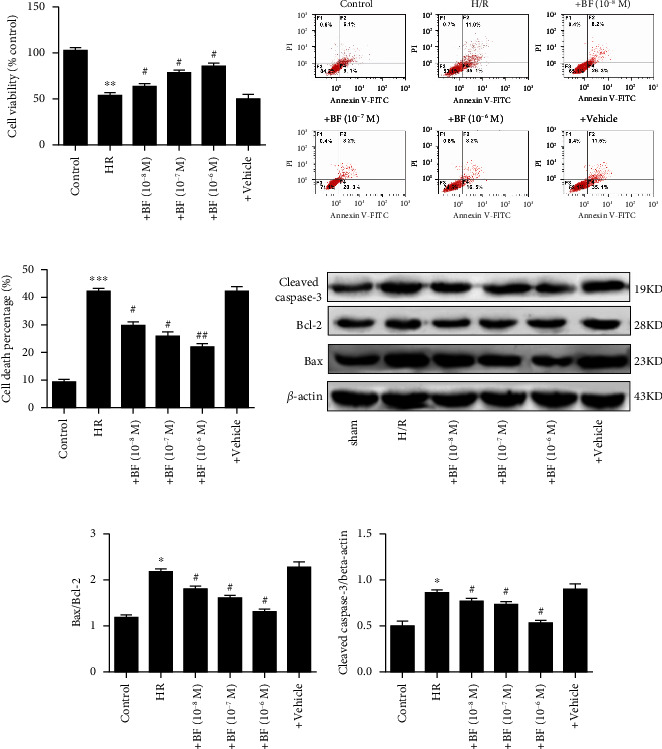
Benzyl ferulate inhibits cell apoptosis of SH-SY5Y cells. (a) Cell viability; (b) representative images of flow cytometry; (c) cell death percentage; (d) protein expression level; (e) the ratio of Bax to Bcl-2; (f) the ratio of cleaved caspase-3 to beta-actin. The differences of the values between groups were analyzed by one-way ANOVA. All experiments were repeated 3 times, and all data are presented as the mean ± standard deviation. H/R: cells subjected to 4-h hypoxia followed by 20-h reoxygenation; +BF (10^−8^ *μ*M): cells subjected to H/R treated with benzyl ferulate (10^−8^ *μ*M); +BF (10^−7^ *μ*M): cells subjected to H/R treated with benzyl ferulate (10^−7^ *μ*M); +BF (10^−6^ *μ*M): cells subjected to H/R treated with benzyl ferulate (10^−6^ *μ*M); +vehicle: cells subjected to H/R treated with DMSO. ⁣^∗^*p* < 0.05 vs control; ⁣^∗∗^*p* < 0.01 vs control; ⁣^∗∗∗^*p* < 0.001 vs control; ^#^*p* < 0.05 vs H/R; ^##^*p* < 0.01 vs H/R.

As shown in Figure 4(a), compared with the control, the cell viability of SH-Y5Y cells obviously reduced after H/R treatment and benzyl ferulate can dose-dependently increase the cellular viability of SH-Y5Y cells are that subjected to H/R treatment. The apoptosis assay by flow cytometry showed that the number of cell deaths obviously increased in SH-Y5Y cells that are subjected to H/R treatment when compared to the control, while benzyl ferulate dose-dependently decreased the percentage of cell death of H/R-treated SH-Y5Y cells (Figures 4(b) and 4(c)). The ratio of Bax/Bcl-2 and the expression of cleaved caspase-3 obviously increased in H/R-treated SH-Y5Y cells and benzyl ferulate dose-dependently decreased the expression of these apoptotic proteins (Figures 4(d), 4(e), 4(f)).

### 3.5. Effect of Benzyl Ferulate on Oxidative Stress in H/R-Treated SH-SY5Y Cells

**Figure 5:**
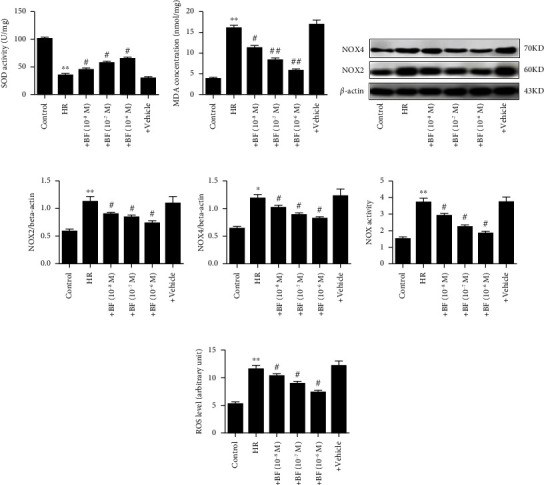
Effect of benzyl ferulate on oxidative stress in H/R treated SH-SY5Y cells. (a) SOD activity; (b) MDA content; (c) protein expression level; (d) the ratio of NOX2 to beta-actin; (e) the ratio of NOX4 to beta-actin; (f) total NOX enzyme activity; (g) ROS level. The differences of the values between groups were analyzed by one-way ANOVA. All experiments were repeated 3 times, and all data are presented as the mean ± standard deviation. H/R: cells subjected to 4-h hypoxia followed by 20-h reoxygenation; +BF (10^−8^ *μ*M): cells subjected to H/R treated with benzyl ferulate (10^−8^ *μ*M); +BF (10^−7^ *μ*M): cells subjected to H/R treated with benzyl ferulate (10^−7^ *μ*M); +BF (10^−6^ *μ*M): cells subjected to H/R treated with benzyl ferulate (10^−6^ *μ*M); +vehicle: cells subjected to H/R treated with DMSO. ⁣^∗^*p* < 0.05 vs control; ⁣^∗∗^*p* < 0.01 vs control; ^#^*p* < 0.05 vs H/R; ^##^*p* < 0.01 vs H/R.

As shown in Figures 5(a) and 5(b), the MDA content was obviously increased and the SOD activity obviously reduced in SH-Y5Y cells that insulted by H/R treatment when compared to the control, while benzyl ferulate dose-dependently reversed these changes by decreasing the MDA content and increasing the SOD activity. The expression level of NOX2 and NOX4, the NOX activity, and the ROS level significantly increased in SH-Y5Y cells that undergone by H/R treatment when compared with the control, while benzyl ferulate dose-dependently reversed these changes by decreasing the expression of NOX2 and NOX4, the total NOX activity, and the ROS level (Figures 5(c), 5(d), 5(e), 5(f), 5(g)).

### 3.6. Effect of Benzyl Ferulate on miRNA Expression in the Brain of CI/R Injury Rats or in H/R-Treated SH-SY5Y Cells

**Figure 6:**
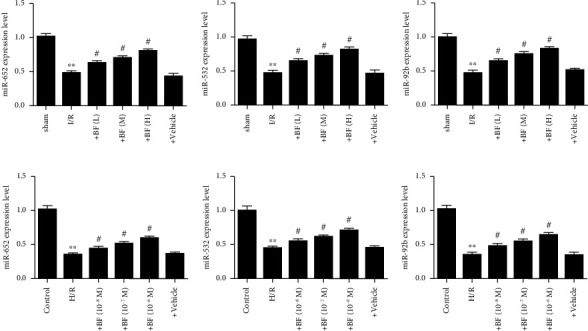
Effect of benzyl ferulate on the expression level of miRNAs in the brain of CI/R injury rats or in H/R treated SH-SY5Y cells. (a) miR-652 expression level; (b) miR-532 expression level; (c) miR-92b expression level; (d) miR-652 expression level; (e) miR-532 expression level; (f) miR-92b expression level. The differences of the values between groups were analyzed by one-way ANOVA. All experiments were repeated 3 times, and all data are presented as the mean ± standard deviation. H/R: cells subjected to 4-h hypoxia followed by 20-h reoxygenation; +BF (10^−8^ *μ*M): cells subjected to H/R treated with benzyl ferulate (10^−8^ *μ*M); +BF (10^−7^ *μ*M): cells subjected to H/R treated with benzyl ferulate (10^−7^ *μ*M); +BF (10^−6^ *μ*M): cells subjected to H/R treated with benzyl ferulate (10^−6^ *μ*M); +vehicle: cells subjected to H/R treated with DMSO. ⁣^∗∗^*p* < 0.01 vs control; ^#^*p* < 0.05 vs H/R.

Compared with the sham, the expression levels of miR-652, miR-532, and miR-92b were obviously downregulated in MCAO rats and benzyl ferulate dose-dependently reversed their expression levels (Figures 6(a), 6(b), 6(c)). As shown in Figures 6(d), 6(e), 6(f), compared with the control, the expression levels of miR-652, miR-532, and miR-92b were obviously downregulated in SH-SY5Y cells that undergone H/R, while benzyl ferulate obviously upregulated their expression dose-dependently.

## 4. Discussion

In this study, we found that benzyl ferulate effectively alleviated brain infarction and relieved apoptosis in the brain of MCAO rats or cell death in SH-SY5Y cells underwent H/R injury by downregulating the expression level of apoptosis-related proteins (Bcl2, Bax) and oxidative stress-related protein (NOX2 and NOX4) and by upregulating the expression level of miRNAs (miR-652, miR-532, and miR-92b). This indicated that benzyl ferulate is a potential antioxidant for ischemic stroke treatment.

It is well known that the imbalance between ROS generation and ROS scavenge contributes to CI/R oxidative stress injury [1]. Studies have demonstrated that antioxidation enzyme activity decreased and oxidative stress increased in acute ischemic stroke patient's serum and that dietary vitamin C intake was negatively related to the incidence of total stroke and ischemic stroke among nonsmokers [14, 15]. This indicated that the inhibition of ROS generation or promotion of ROS scavenges is an effective way for neuroprotection theoretically. Cumulating studies have shown that antioxidants, such as apocynin, vitamin C, vitamin E, resveratrol, allopurinol, and *N*-acetylcysteine, could prevent brain injury in vitro [16–18]. These data fully demonstrated that the antioxidation strategy is effective for ischemic stroke therapy. In this study, obvious injuries occurred in MCAO rats and in H/R-treated SH-Y5Y cells were indicated, such as impaired neurobiological function, brain infarction, and cell death or apoptosis. In addition, the present data showed that the MDA content obviously increased while the SOD activity reduced in brain tissues in MCAO rats or in H/R-treated SH-SY5Y cells. This indicated that there is obvious oxidative stress occurred in both the MCAO rat model and the H/R-treated SH-SY5Y cell model and that both are feasible models for oxidative stress injury study. As NOX is one of the leading enzymes for ROS production during the process of CI/R injury and NOX2 and NOX4 are the main subtypes of NOX for ROS production in CI/R injury [7, 8], thus NOX2 and NOX4 are important targets for oxidative stress injury studies, such as antioxidant drug development. In fact, previous studies have shown that drugs that target NOX2 or NOX4 effectively alleviated the oxidative stress injury of the brain tissues of MCAO rats, such as apocynin, VAS2870, gp91ds-tat, and GKT137831 [17, 19, 20]. In the present study, the findings showed that NOX2 and NOX4 expression, the total NOX activity, and the ROS level are significantly increased. These data indicated that NOX2 and NOX4 are potential targets for the screen of antioxidant drug using for ischemic stroke therapy.

For decades, finding antioxidants from traditional Chinese medicine has received the attention of researchers [21–23]. Among them, the constituent extracts from *Ligusticum chuanxiong Hort*., such as ferulic acid, proved to be neuroprotective in CI/R injury via antioxidation [10, 21]. Therefore, ferulic acid is an important parent drug for the treatment of CI/R injury. Benzyl ferulate, a derivative of ferulic acid, may be easier to reach brain tissue than ferulic acid due to its higher lipid solubility. This was proved by the result that the concentrations of benzyl ferulate in plasma and brain tissue homogenates were 4.5 *μ*g/mL and 0.45 *μ*g/g, respectively, which were significantly higher than those of ferulic acid (2.75 *μ*g/mL and 0.24 *μ*g/g) (Figure S1). Thus, we speculated that benzyl ferulate also has antioxidation action theoretically, which was confirmed by the results that benzyl ferulate attenuated brain infarction size of rats via inhibiting the expression of NOX2 and NOX4. Further studies proved that these inhibitions were associated with the expression regulation of miR-652, miR-532, and miR-92b, which are targeted miRNAs of NOX2 or NOX4. As shown in Figure S2, following CI/R injury, the expression of miRNAs (miR-652, miR-532, and miR-92b) in brain tissue is downregulated, resulting in the upregulation of their target genes NOX2 and NOX4, which leads to excessive ROS generation and eventually nerve cell death (Figure S2). This suggests that drugs that involved in the expression regulation of miRNAs (miR-652, miR-532, and miR-92b) may have a potential antioxidant effect. It is shown that the benzyl ferulate can upregulate the expression level of miR-652, miR-532, and miR-92b in MCAO rats or in H/R-treated SH-SY5Y cells in a dose-dependent manner. This hints that antioxidant drugs that inhibiting NOX or regulating the expression level of miRNAs-targeted NOX (especially NOX2 and NOX4) are candidates for CI/R injury therapy. It has also been confirmed by previous studies that VS2870, a NOX inhibitor, can improve outcomes of rats after CI/R injury via increasing the expression of miRNAs (miR-29, miR-126 and miR-132, miR-92b) that are potentially targeted miRNAs of NOX2 or NOX4 [24, 25]. These data above indicated that benzyl ferulate is a promising candidate for antioxidant for CI/R oxidative stress injury.

Although the antioxidation of benzyl ferulate was found in our in vitro or in vivo models, more works need to be done before its clinical use or even extensive pharmacological research, such as pharmacokinetics studies and toxicological studies. In addition, besides it involving in the regulation of NOX2 and NOX4 in CI/R injury, whether benzyl ferulate involves in other enzymes, such as xanthine and SOD, that still need more works.

Collectively, benzyl ferulate attenuated the CI/R oxidative stress injury via the expression regulation of miRNAs (miR-652, miR-532, and miR-92b)/NOX2/NOX4 axis that mainly related to ROS generation in mitochondria in neuron cells. This study first proved that benzyl ferulate is a potential antioxidant for ischemic stroke therapy.

## Data Availability

The dataset used and/or analyzed during the current study are available from the corresponding author upon reasonable request.

## Supporting Information

The supporting information containing two figures, the one is about the concentrations of ferulic acid and benzyl ferulate in plasma and brain tissue homogenates (Figure S1) and the other is a schematic diagram about miRNA (532/652/92b) involves in the expression regulation of NOX2 and NOX4 in cerebral ischemia/reperfusion injury (Figure S2).
