# Supplementary material for: Neuroprotective Effect of Benzyl Ferulate on Ischemia/Reperfusion Injury via Regulating NOX2 and NOX4 in Rats: A Potential Antioxidant for CI/R Injury
Source: Adv Pharmacol Pharm Sci. 2024 Nov 2;2024:5534135. doi: 10.1155/2024/5534135 (PMC11550003; doi:10.1155/2024/5534135)
Supplement: Supporting Information — Additional supporting information can be found online in the Supporting Information section. [file 5534135.f1.docx]

***FigureS1. The concentrations of ferulic acid and benzyl ferulate in plasma and brain tissue homogenates***

As shown in figureS1, higher concentrations of benzyl ferulate in plasma(4.5μg/ml) and in brain tissue homogenates (0.45μg/g) than that of ferulic acid(counterparts were 2.75μg/ml and 0.24μg/g, respectively). This suggested that benzyl ferulate with higher bioavailability than ferulic acid.

A. the concentrations of ferulic acid and benzyl ferulate in plasma;B the concentrations of ferulic acid and benzyl ferulate in brain tissue homogenates. All experiments were repeated 3 times and all data are presented as the mean ±standard deviation. FA:felulic acid(20mg/kg weight); BF: benzyl ferulate (20mg/kg weight).**P＜0.01 vs FA.


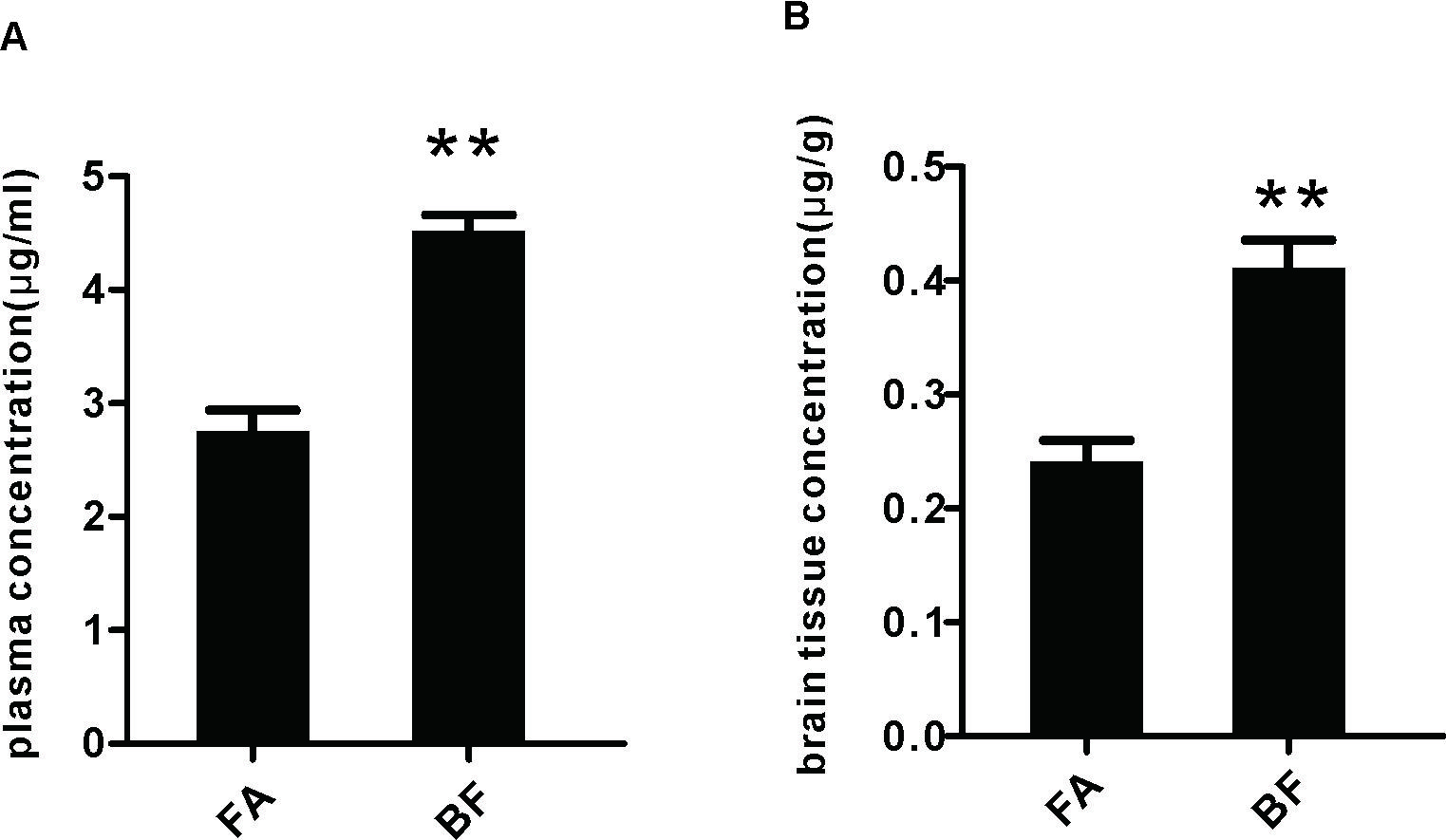


FigureS1

***FigureS2. miRNA(532/652/92b) involves in the expression regulation of NOX2 and NOX4 in cerebral ischemia/reperfusion injury***

As the schematic diagram shown, following cerebral ischemia-reperfusion injury, the expression of miRNAs(miR-652, miR-532 and miR-92b) in brain tissue is down-regulated, resulting in the upregulation of their target genes NOX2 and NOX4, which leads to excessive ROS generation and eventually nerve cell death.


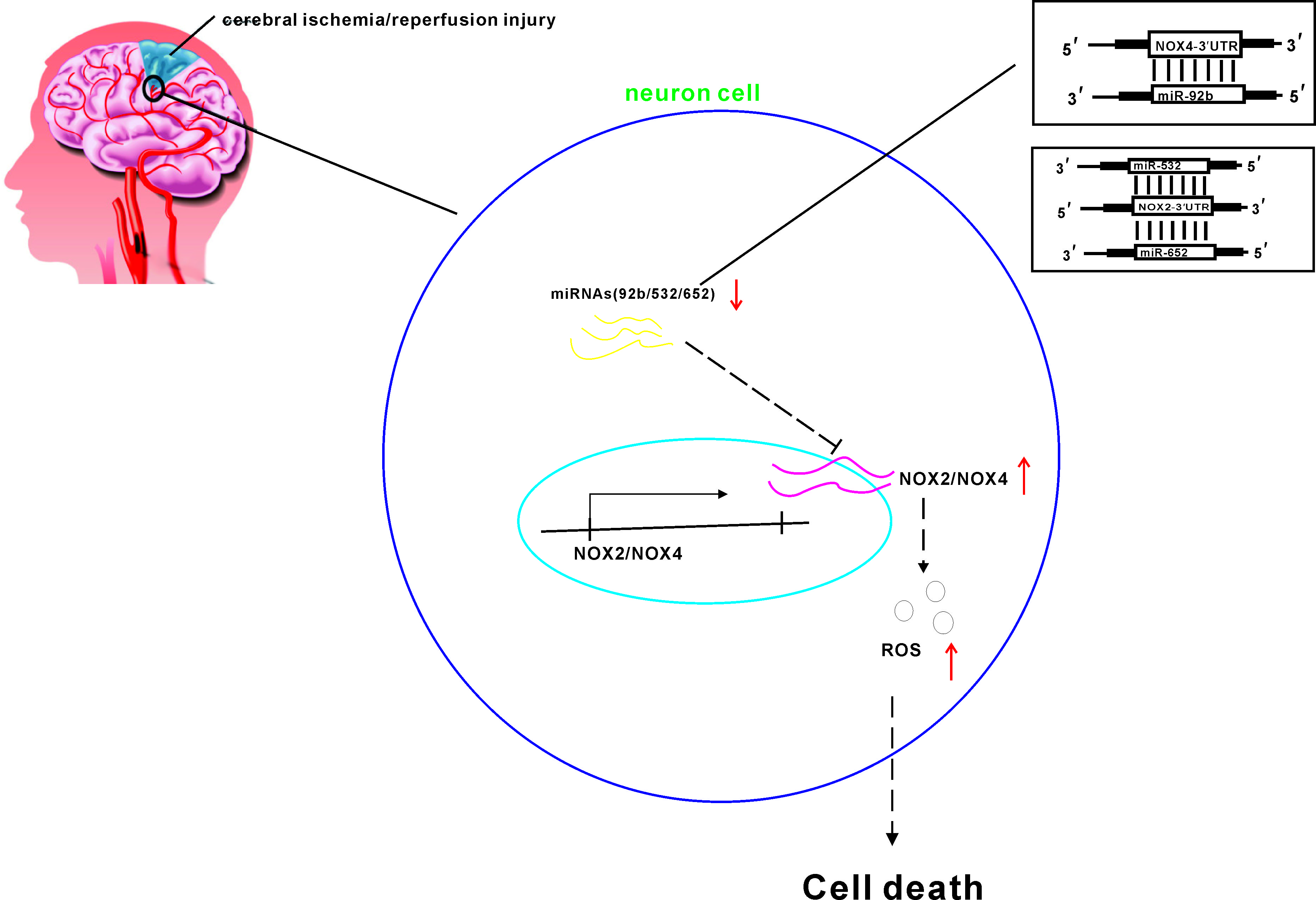


FigureS2
